# Impact of adsorption on thermal conductivity dynamics of adsorbate and adsorbent: Molecular dynamics study of methane and Cu-BTC

**DOI:** 10.1016/j.isci.2024.110449

**Published:** 2024-07-04

**Authors:** Haonan Chen, Sagar Saren, Xuetao Liu, Ji Hwan Jeong, Takahiko Miyazaki, Young-Deuk Kim, Kyaw Thu

**Affiliations:** 1 Department of Advanced Environmental Science and Engineering, Faculty of Engineering Sciences, Kyushu University, Kasuga-koen 6-1, Kasuga, Fukuoka 816-8580, Japan; 2 Institute of Innovation for Future Society, Nagoya University, Furu-cho, Chikusa, Nagoya, Aichi 464-8603, Japan; 3 School of Mechanical Engineering, Pusan National University, Geumjeong-gu, Busan 46241, South Korea; 4 Research Center for Next Generation Refrigerant Properties (NEXT-RP), International Institute of Carbon-Neutral Energy Research (WPI-I2CNER), Kyushu University, 744 Motooka, Nishi-ku, Fukuoka, Fukuoka 819-0395, Japan; 5 BK21 FOUR ERICA-ACE Center, Hanyang University, 55 Hanyangdaehak-ro, Sangnok-gu, Ansan, Gyeonggi-do 15588, Republic of Korea; 6 Department of Mechanical Engineering, Hanyang University, 55 Hanyangdaehak-ro, Sangnok-gu, Ansan, Gyeonggi-do 15588, Republic of Korea

**Keywords:** Engineering, Materials science, Computational materials science

## Abstract

Understanding changes in thermodynamic and transport properties during adsorption is crucial for the thermal management of metal-organic frameworks, which also imposes significant challenges for improved performance and energy density of adsorption system. Because of phase transitions in the intermolecular interactions involved in the adsorption phenomena, transport properties including the thermal conductivity exhibit interesting behaviors, yet fully understood. This study employs detailed molecular dynamics simulations to replicate the methane/Cu-BTC adsorption phenomenon for the evaluation of their thermal conductivities across different pressures and temperatures. The molecular simulations show that the thermal conductivities of both the adsorbent (Cu-BTC) and adsorbate (methane, adsorbed phase) vary notably during adsorption processes. Using the concepts of the change in the degree of free movements of the adsorbate molecules and atomic vibration of adsorbent, the reduction of the adsorbate thermal conductivity (∼70–93%) and increase thermal conductivity of the adsorbent (up to 3 times) in Cu-BTC+CH_4_ pair are explained.

## Highlights

• Modeled methane and Cu-BTC adsorption by molecular dynamics from the very beginning
• Calculated the thermal conductivity for both adsorbate and adsorbent
• Explained the thermal conductivity variation in the atomic scope
• Provided an insight into microscopic adsorption phenomena

## Graphical abstract

**Figure:**
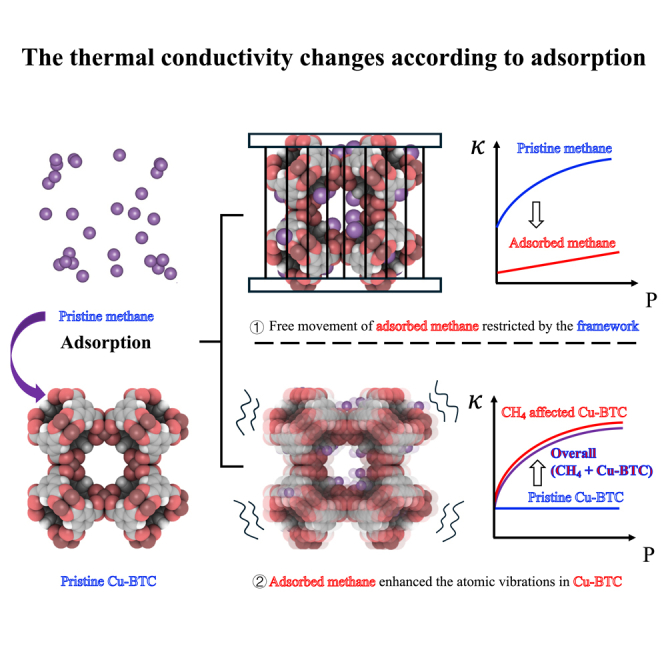

## Introduction

Metal-organic frameworks (MOFs), owing to their unique structure, find extensive applications in fields such as carbon capture and storage,^1^^,^^2^ adsorption heat pumps and chillers,^3^^,^^4^ desiccant heat exchangers,^5^^,^^6^ and seawater desalination.^7^ Their thermodynamic properties significantly impact engineering applications. For instance, during the process of gas adsorption and desorption, the release or absorption of isosteric heat alters the adsorbate’s quantity and rate of adsorption, directly affecting MOF performance. Therefore, research into the thermal properties of MOFs has been rapidly increasing in recent years.^8^^,^^9^ Among these, changes in the thermal conductivity of MOFs after adsorption represent a challenge. Despite the challenges associated with measuring such materials with small volumes, a variety of experiments^10^^,^^11^^,^^12^ and classical molecular dynamics (MD) simulations^13^^,^^14^^,^^15^ have been conducted to explore their thermal conductivities. Attempts have been expanded to include the effects of pore size and defects within MOFs.^16^^,^^17^ Moreover, understanding how adsorbates influence the thermal conductivity of MOFs remains a significant and contributory area of study.

Few researchers observed that the adsorption loading increased the overall thermal conductivity of MOF. Huang et al.^18^ measured the thermal conductivity of compressed MOF powder: UiO-66, UiO-67, and Cu-BTC with various water contents by transient hot-wire method. They found that the MOF powder thermal conductivity significantly increased with the increase in the water content because the occupation of water in the MOF reduced the inter-particle resistance. Cui et al.^19^ used the transient heat source technique to investigate the thermal conductivity of MIL-160(Al) with water adsorption. At the ambient pressure, the thermal conductivity of MOF was 0.065 W/mK. After the adsorption of water from ambient pressure to 25.91kPa, the thermal conductivity rose from 0.071 to 0.117 W/mK. Han et al.^20^ studied hydrogen and deuterium adsorption in MOF-5 using MD simulation. The result suggested that the MOF thermal conductivity increases with the gas load and gas mass. In their perspective, the adsorbed gas provided an additional heat transfer channel from MOF nodes to linker, due to the additional disorder and scatter caused by adsorbed gas. Wei et al.^21^ also had a similar finding by MD for the ethanol and water adsorption in ZIF-8. Both water and ethanol adsorption improved the thermal conductivity of ZIF-8, but ethanol exhibited a stronger enhancement. The vibrational density of states showed that ethanol vitally strengthened the vibration of Zn and N atoms of ZIF-8, thus its thermal conductivity increased remarkably. Babaei et al.^22^ discussed the anisotropy of thermal conductivity toward the CO_2_ adsorption in e−2 appended Zn2(dobpdc). They declared that the cooperative formation of metal-bound ammonium carbamates became an additional heat transfer pathway to increase the thermal conductivity along the pore channel. Not only in MOF, but covalent organic frameworks (COF), zeolite, and activated carbon also have the tendency of thermal conductivity increase during the adsorption process.^23^^,^^24^^,^^25^

On the contrary, there is other research supporting that the load of adsorbate decreased the overall thermal conductivity of MOF. Babaei et al.^26^ adopted frequency-domain thermoreflectance (FDTF), time-domain thermoreflectance (TDTF), and MD Green-Kubo method to evaluate the thermal conductivity change during the adsorption of water, methanol, and ethanol on Cu-BTC. The results certified that the thermal conductivity of Cu-BTC decreased 40–80%, which was caused by phonon scattering. Fan et al.^27^ used non-equilibrium molecular dynamics (NEMD), and also agreed that when water was adsorbed on Cu-BTC, the thermal conductivity reduced up to 51.3%. However, with the increase in the amount of water adsorbate, the thermal conductivity of Cu-BTC decreased first, then increased. This phenomenon was suggested as a competition between the phonon scattering of MOF and the heat transfer of water molecules. Purewal et al.^28^ analyzed H_2_ adsorption on MOF-5 tablets by a commercial xenon flash thermal diffusivity instrument. They found that although the load of H_2_ increased not only the density but also the thermal conductivity of MOF-5, yet still lower than MOF-5 single crystal. At cryogenic temperature, Schlemminger et al.^29^ measured the thermal conductivity of Fe-BTC with the adsorbate of He, N_2_, and Ar by transient plane source method. Similarly, they concluded all the thermal conductivity of Fe-BTC with adsorbate was lower than Fe-BTC single crystal, and indicated that the thermal conductivity had strong temperature dependency in the range of 77–400 K.

Although there is still debate regarding the change in thermal conductivity of MOFs before and after the adsorbate loading, the attention to how the understanding of the thermal conductivity variations of the adsorbent and the adsorbate during the adsorption process is limited. It remains a mystery whether it was possible to measure the thermal conductivities of the adsorbent and adsorbate separately within an experiment simultaneously. In this paper, we study the thermal conductivity change for each component due to the adsorption, using classical MD, with the adsorption pair of methane and Cu-BTC. The MD simulations in this article are divided into three parts: the adsorption kinetics of methane on Cu-BTC; the calculation of the overall thermal conductivity of the methane/Cu-BTC system, as well as that of each component; and finally, the calculation of the thermal conductivity of pristine Cu-BTC crystals, and the pristine adsorbed phase methane. We validated the simulation results with the experimental data available in the literature. The molecular simulations showed that the thermal conductivities of the adsorbent (Cu-BTC) and adsorbate (adsorbed phase) vary notably during adsorption processes. Using the concepts of the change in the degree of freedom (restrictions of the free movements of the adsorbate) of the adsorbate molecules and atomic vibration, we attempted to explain the reduction in the adsorbate thermal conductivity and increase in the thermal conductivity of the adsorbent.

## Result

The results section is systematically divided into four distinct subsections. The initial subsection delineates the simulation results of methane adsorption, which serves to corroborate the dependability of intermolecular force fields predicated upon Lorentzian formulations for the DREIDING and UFF. This verification is critical to ensure the proximity of the adsorption simulations to authentic scenarios. The second subsection explicates the simulated data for both pristine methane and pristine Cu-BTC. For methane, comparisons were drawn with NIST benchmarks to ascertain the fidelity of the force field. The thermal conductivities of the adsorbed phase of methane, deduced via the GK method and the Einstein method from the On-the-fly calculation of Transport Properties (OCTP) plugin, were juxtaposed against NIST values to affirm the precision of the GK methodology. The GK method was further utilized to gauge the thermal conductivities of Cu-BTC, the results of which were instrumental in establishing the trustworthiness of the MOF-FF.

In the last two subsections, the point of discussion is the influence of adsorption on methane’s thermal conductivity, where the mean free path has been invoked as a qualitative indicator to evaluate the mechanism of the observed thermal conductivity variations. The last subsection broaches the subject of adsorption’s effects on the thermal conductivity of Cu-BTC, employing the phonon density of states (PDOS) and lattice mean displacement (LMD) as qualitative tools to shed light on the causal factors influencing the thermal conductivity alterations.

### Methane adsorption analysis

**Figure 1:**
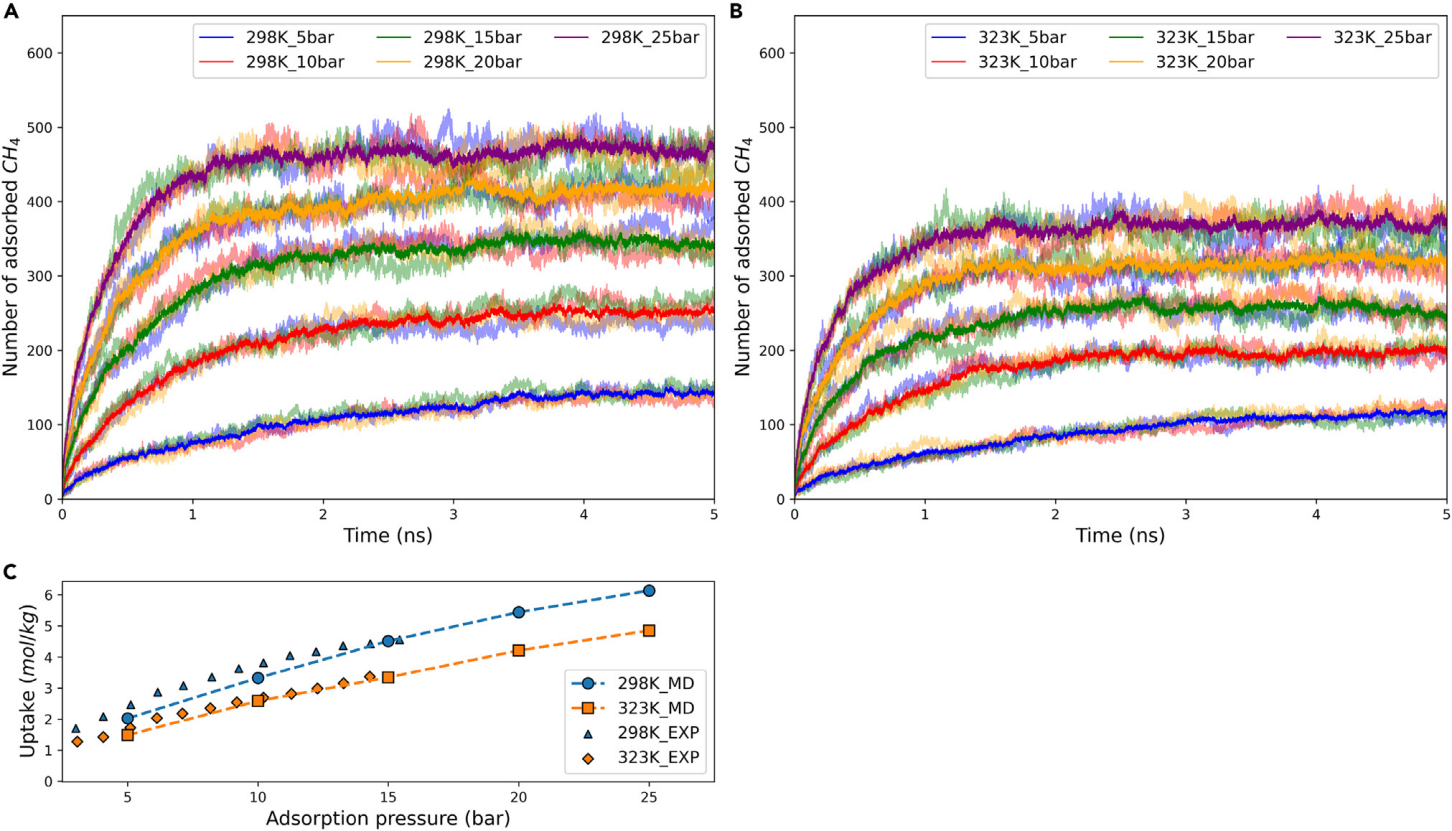
Methane adsorption verification (A) The adsorption kinetics of methane/Cu-BTC at 298K. (B) The adsorption kinetics of methane/Cu-BTC at 323K. (C) The isotherm of methane/Cu-BTC adsorption.

In the adsorption simulation process, ambient gaseous methane molecules undergo Brownian motion within the simulation box with periodic boundary conditions. However, molecules near the Cu-BTC clustered on the surface or entered the framework due to van der Waals forces. The Cu-BTC model is positioned at the center of the simulation box to ensure that CH_4_ molecules from all directions have equal opportunities to enter the MOF. Once methane adsorption within the framework reached saturation, the adsorption kinetics curve reached a dynamic equilibrium, indicating the completion of adsorption. Figures 1A and 1B display the adsorption kinetics up to 5 ns under various pressures at 298K and 323K. The lighter lines represent individual simulation outcomes, while the darker lines indicate the averages. For each curve, the adsorption rate is initially significant and then decreases to a dynamic equilibrium, in agreement with experimental observations. Higher pressures facilitate methane adsorption, evident from the shortened adsorption time and increased equilibrium adsorption capacity. Upon comparing the two figures, it is observed that higher ambient temperatures could also shorten the adsorption time, although the equilibrium adsorption capacity is reduced. This agrees with the physical properties indicating that high temperatures facilitate desorption. Furthermore, at 25 bar, significant fluctuations are observed in each independent simulation. Multiple simulations were essential to minimize the uncertainty. After computing the average number of methane molecules adsorbed in the last 1 ns under all conditions, the adsorption isotherm is presented in Figure 1C. When compared to the experimental results of methane adsorption in Cu-BTC measured by Liang et al. using an intelligent gravimetric analyzer (IGA),^30^ the current simulated adsorption isotherm corresponds closely with the experimental data at 323K. However, the largest discrepancy is observed at 298K, 5 bar with an error of 18.66%, but still within the allowable range. Hence, the intermolecular force field between methane and Cu-BTC can be considered reliable.

### Pristine methane and pristine Cu-BTC properties

**Figure 2:**
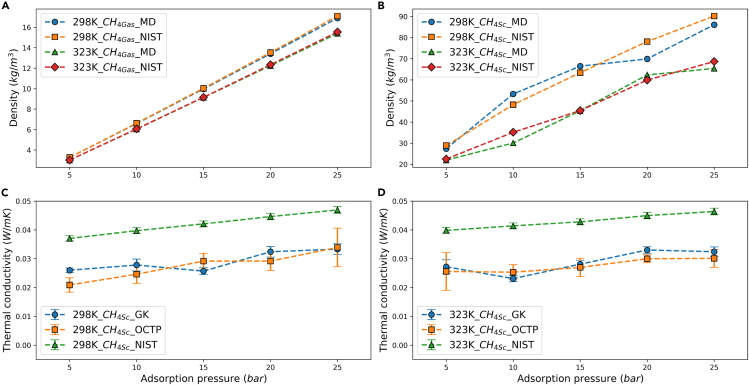
Methane property verification (A) The density verification of gas phase methane. (B) The density verification of supercritical methane. (C) The thermal conductivity verification of supercritical methane at 298K (Data are represented as mean ± SEM). (D) The thermal conductivity verification of supercritical methane at 323K (Data are represented as mean ± SEM).

In the simulation of methane adsorption, the initial step involves the creation of a sufficiently large gaseous methane environment. During this process, the temperature, pressure, and density of gaseous methane are recorded. Figure 2A illustrates a comparison of the simulated density of gaseous methane under various conditions compared to the data from NIST. The results are in close agreement, with a maximum discrepancy of 1.14%. A similar approach was employed for the simulation of the adsorbed phase methane. In the simulations, due to the adsorption of methane resembling a condensation process, a concentration amplification characteristic was observed, which means the density of the adsorbed methane was higher than the gaseous phase. Put the same amount of adsorbed phase methane in an empty simulation box of the same size, the calculated pressures range from 35 bar to 116 bar. Compared to the gaseous adsorption pressure, adsorption results in a concentration amplification effect of 4–8 times, without considering the volume occupied by Cu-BTC. According to NIST data, excluding the volume of Cu-BTC, the adsorbed phase methane remains gaseous only at an adsorption pressure of 5 bar, while at all other pressures, it is in the supercritical state. To differentiate it from the methane components of the methane/Cu-BTC composite system in the subsequent sections, the pristine adsorbed phase methane was referred to as the supercritical methane in the following content, while the methane components of the methane/Cu-BTC composite system were termed, here, as the adsorbed phase methane. Figure 2B presents a comparison of the simulated density of the supercritical methane with NIST data, where the X-axis corresponds to the adsorption pressure of the gaseous methane. The simulation results are broadly consistent with NIST data, with a mean absolute error of 6.15% and a maximum absolute error of 14.54% at 323K and 10 bar.

For the supercritical methane, its thermal conductivity was calculated using the traditional GK method and the Einstein relation by OCTP plugin. Figures 2C and 2D show the comparison results between these two methods and NIST data. The GK and OCTP results were similar, with a mean absolute error of 9.21%. The convergence of the results calculated from different algorithms to some extent illustrates the commonality of the molecular force field. Compared to NIST data, the calculated thermal conductivities show the same trend with increasing adsorption pressure but differ significantly in value, with mean absolute errors of 32.25% and 35.51% in Figures 2C and 2D, respectively. This discrepancy can be attributed to the TraPPE methane molecule being a coarse-grained model, simplifying a five-atom molecule into a single-atom model to increase computational speed while reducing the degrees of freedom. Consequently, the impact of interatomic bond energy and the vibration contribution of each atom on thermal conductivity cannot be fully accounted for, thus marginally reducing the accuracy of the calculated thermal conductivity.^31^ Nonetheless, due to the relative stability of the error, the force field was able to adequately describe the thermal conductivity of methane. The GK method, being highly susceptible to sampling randomness, does not show a monotonic increase in thermal conductivity with increasing pressure at the same temperature, which is common in simulations of non-solid small systems.^32^ The OCTP method, utilizing an N-stage algorithm for improved sampling, reflected the same monotonic increasing trend, but is theoretically more suited for simulations of liquid systems,^33^ and thus, was only used for calculating the supercritical phase thermal conductivity in this study. The analysis of methane density and thermal conductivity confirms the TraPPE methane force field’s capability to reflect its physical properties accurately.

For the pristine Cu-BTC, the thermal conductivity at 298K and 323K was calculated using the GK method to be 0.5799 ± 0.0225 W/(mK) and 0.4969 ± 0.0235 W/(mK), respectively. These values are consistent with the range measured by Babaei et al. using the TDTR method for Cu-BTC thin films, which varied from 0.44 to 0.73 W/(mK).^26^ Thereby the reliability of the MOF-FF is validated.

### Effect of adsorption on methane’s thermal conductivity

**Figure 3:**
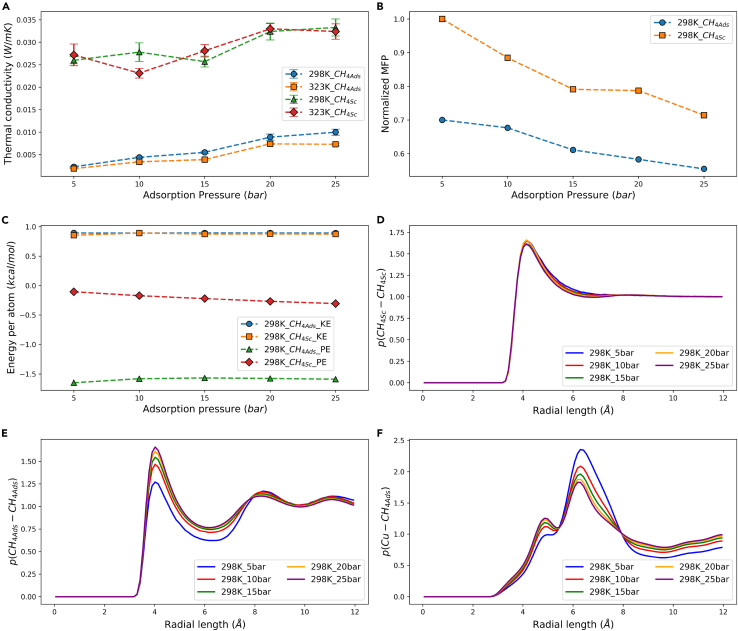
Thermal conductivity profiles and relevant investigation of methane with adsorption Adsorbed phase and supercritical methane (A) thermal conductivity (Data are represented as mean ± SEM), (B) normalized mean free path, (C) kinetic and potential energy. The radial distribution function of (D) CH_4_-CH_4_ in the supercritical phase, (E) CH_4_-CH_4_ in the adsorbed phase, (F) Cu-CH_4_ in the methane/Cu-BTC composite system.

In the previous section, it was discovered that the thermal conductivity of the supercritical methane exhibited an increasing trend with the rise in adsorption pressure. Figure 3A illustrates the changes in thermal conductivity for both supercritical and adsorbed phase methane. It is evident that the thermal conductivity of the adsorbed phase methane is significantly lower than that of supercritical methane, reducing around 70–93%. However, similar to the supercritical state, its thermal conductivity also increases with the rise in the adsorption pressure. To investigate this phenomenon, the mean free path was calculated for all conditions at 298K. The mean free path did not change significantly under a small temperature differential, so it was not calculated at 323K. For the ease of comparison, the mean free path of supercritical methane at 298K and 5 bar was taken as the baseline, and other results were normalized against it. As observed in Figure 3B, the mean free paths of both supercritical and adsorbed phase methane decrease with increasing adsorption pressure, but the mean free path of adsorbed phase methane remains lower than that of the supercritical state. Gases and liquids primarily transfer heat through molecular collisions, and a higher density promotes this process. Hence, there is a tendency for the thermal conductivity to increase with higher adsorption pressure. A higher density results in more frequent molecular collisions, so the mean free path decreases, which aligns well with the current simulation results. However, this cannot explain why the thermal conductivity of the adsorbed phase is lower than that of the supercritical state. In the simulations of the adsorbed phase methane, MOFs occupy a portion of the volume, and theoretically, the density is higher than that in simulations of the supercritical methane. The lower mean free path depicts in the figure reflected this scenario.

Subsequently, our presumption is that the Cu-BTC framework captures a portion of methane, restricting its free movement and thus lowering its thermal conductivity. To substantiate this hypothesis, the energy and radial distribution function of methane in the simulations were examined. In large-scale atomic/molecular massively parallel simulator (LAMMPS), the calculation of heat flux consists of two components: energy and tensor; energy included kinetic and potential energies, while tensor includes kinetic and virial contributions.^34^ Therefore, heat flux involves three physical quantities: kinetic energy, potential energy, and virial contribution. Molecular kinetic energy is determined solely by the system temperature. As the temperature set the same for the supercritical and the adsorbed phase simulation, the methane kinetic energies in both types of simulations are expected to be very close, and indeed they are (see Figure 3C). Therefore, kinetic energy is not the key factor. Potential energy and virial contribution, which are physical quantities that describe intermolecular forces, are highly similar. Here, only potential energy is chosen for discussion. Figure 3C indicates that the potential energy of adsorbed phase methane is much lower than that of supercritical methane. This suggests that adsorbed phase methane is a more stable entity, and according to the calculation formula, the heat flux value has been reduced. Although the GK method uses fluctuations of heat flux to calculate thermal conductivity, a reduction in the absolute value would directly affect the amplitude, thus explaining the reduced thermal conductivity of the adsorbed phase. Surface adsorption is the most direct process for potential energy reduction, similar to the principle of Grand Canonical Monte Carlo (GCMC).^35^ Therefore, this served as indirect evidence of methane being captured by the framework.

Figure 3D shows the radial distribution function of supercritical methane at 298K under different pressures, with only one peak within the cutoff distance, which is typical of a gaseous state. It can be seen that with increasing pressure, the change in the peak’s height is not significant, but its width becomes narrowed, indicating an increase in the system’s orderliness, consistent with the results of increasing thermal conductivity.

Figure 3E presents the radial distribution function of adsorbed phase methane at 298K under different pressures, composed of three peaks, which at first glance appears to be a typical liquid state. However, this is not due to a phase change but rather the presence of the MOF framework altering the distribution of methane, with adsorbed phase methane still closely resembling a gaseous state. The primary peak height in Figure 3E, similar to Figure 3D, does not increase significantly, such as 2 or 3 times, which further supports our hypothesis. Thus, after methane was adsorbed into Cu-BTC, its movement pathways were indeed affected, and this impact was a form of restriction, as the local density formed three regular peaks. Moreover, the primary peak height increases noticeably with increasing adsorption pressure, indicating a higher concentration of methane, which supports the result of the increased thermal conductivity. At the same time, the primary peak height at 10 bar was significantly higher compared to 5 bar, which did not suggest a substantial increase in thermal conductivity but indicated a notable change in methane distribution within the MOF framework.

**Figure 4:**
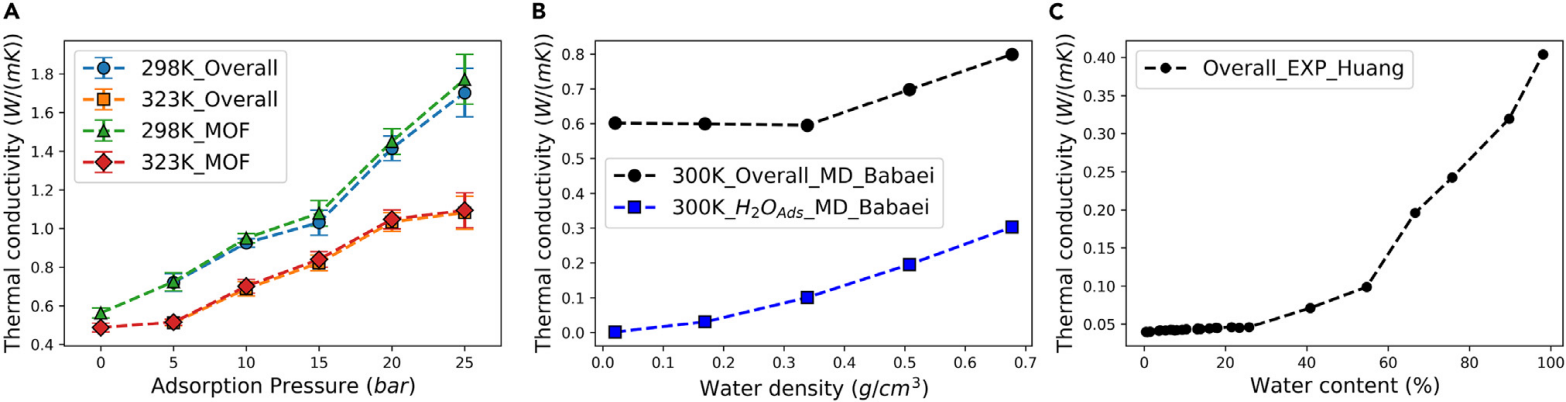
Thermal conductivity profiles with different adsorption pairs (A) the thermal conductivity of methane/Cu-BTC overall and Cu-BTC component (Data are represented as mean ± SEM), (B) the thermal conductivity of water/Cu-BTC overall and adsorbed phase water component by Babaei et al.^26^ (C) the thermal conductivity of water/Cu-BTC overall by Huang et al.^18^

Figure 3F depicts the radial distribution function of the Cu atoms of Cu-BTC to methane molecules in the methane/Cu-BTC composite system, revealing that after 5 bar, the first peak on the left gradually forms and is lower than the primary peak’s height, attributable to the structure of Cu-BTC. As a porous material, Cu-BTC consists of two types of cages. From the cell perspective, there is a central main cage and eight peripheral secondary cages. At low pressures, methane primarily occupied the main cage, and as the adsorption pressure increased, it gradually filled the secondary cages. This phenomenon explains the rise and fall of the two peaks in Figure 3F, while also corroborating the result of restricted movement of adsorbed phase methane.^36^ Babaei et al. observed a similar decrease in the thermal conductivity of water adsorbed in Cu-BTC compared to pure water, proposing that the small pore size of Cu-BTC truncated the transport of water, thereby reducing heat transfer^26^ (The details are discussed in Figure 4A). This was akin to the capture hypothesis proposed in this paper.

In conclusion for this subsection, compared to the supercritical methane, the reduced thermal conductivity of methane after adsorption was due to it being captured by the MOF framework, with lowered potential energy, and restricted free movement. Additionally, with the increase in adsorption pressure, the enhanced methane density increased molecular collision opportunities, thus increasing the thermal conductivity.

### Effect of adsorption on Cu-BTC’s thermal conductivity

Figure 4A illustrates the variations in the overall thermal conductivity alongside the thermal conductivity of the Cu-BTC component among the methane/Cu-BTC composite system in this work. As the adsorption pressure increases, both the overall thermal conductivity and that of the Cu-BTC component exhibit a monotonous rise, up to 3 times. Numerically, the overall thermal conductivity represents the average of the thermal conductivity of the adsorbed phase methane and the Cu-BTC component. Since the thermal conductivity of the adsorbed methane is significantly lower than that of the Cu-BTC component, the overall conductivity lies between the two.

Experimental data on the adsorption of methane with Cu-BTC are scarce but our investigation revealed parallel tendencies within the research about the adsorption of water on Cu-BTC. Babaei et al. discovered in their TDFD experiments that water adsorption decreased the thermal conductivity of Cu-BTC, and they replicated the process using MD simulations.^26^ In their findings, the slight presence of water adsorption resulted in an approximate 50% drop in conductivity compared to pristine MOF, which they suggested, was due to phonon scattering. However, with increasing water content, as shown in Figure 4B, the overall thermal conductivity rose, as well as the adsorbed water, where both trends were similar to the results of this study. For the thermal conductivity of pristine Cu-BTC, Babaei’s MD result was close to 1.3 W/mK, significantly higher than their experimental results of 0.4–0.7 W/mK. The simulation result in this work, as mentioned in pristine Cu-BTC properties, was at 0.5799 W/mK, falling within the experimental range, attributed to different molecular force fields used. Figure 4C presents another experimental result. Huang et al. measured the overall thermal conductivity changes in compacted Cu-BTC powder with water adsorption using the classical transient hot-wire method.^18^ It was observed that the thermal conductivity continuously increased with water content. To some degree, the adsorption of water on Cu-BTC supports the notion that adsorption can lead to an increase in overall thermal conductivity.

**Figure 5:**
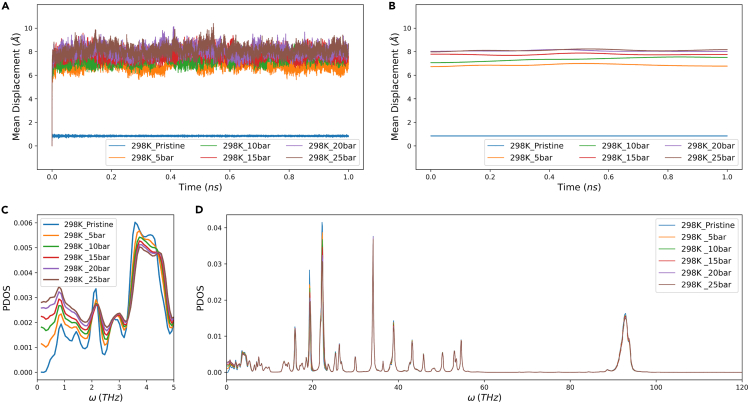
Atomic investigation for Cu-BTC with adsorption (A) the lattice mean displacement of Cu-BTC, (B) the smoothed lattice mean displacement of Cu-BTC, (C and D) the phonon density of states of pristine and methane-adsorbed Cu-BTC.

Such an increase in thermal conductivity is not fully understood and worth investigating. Some literature suggests that the adsorbate becomes an additional thermal channel, enhancing the overall thermal conductivity, akin to a series-parallel additive effect.^20^^,^^37^ We propose a new hypothesis that the inclusion of the adsorbate acts as a catalyst, not to reduce activation energy though, but to enhance the vibrations of the solid framework, since the conduction of heat in solid MOFs mainly arises from atomic vibrations.^38^ To verify this hypothesis, calculations of LMD and PDOS were carried out, analyzing only at 298K. As shown in Figure 5A, the introduction of methane significantly increases the amplitude of LMD, compared to pristine Cu-BTC. Figure 5B which is a Gaussian smoothing of Figure 5A, shows higher LMD with increased adsorption pressure, indicating that adsorbed methane enhanced not only the chaos but also the intensity of the atomic vibrations of Cu-BTC, which corresponds to the increased thermal conductivity observed in Figure 4A. To further illustrate the change in atomic vibrations, the PDOS were calculated in the range of 0–120 THz based on the velocities of the atoms, with a sampling frequency of every femtosecond. Figure 5D displays the overall PDOS, revealing that most phonons are within 0–60 THz. With increasing adsorption pressure, as Figure 5C indicates, apart from the low-frequency range (0–2 THz) where phonon density increases, other peaks decline. Low-frequency phonons contribute the most to thermal conductivity, as high-frequency phonons have shorter lifetimes due to phonon scattering, which is detrimental to heat transfer.^39^ Moreover, the density in the low-frequency range also increased with rising adsorption pressure, supporting that the enhancement of atomic vibrations led to improved thermal conductivity.

In conclusion of this subsection, for Cu-BTC, the addition of adsorbed methane intensified the vibrations of the framework’s atoms, increased the density of low-frequency phonons, and as a result, elevated the thermal conductivity.

## Discussion

Temperature, as a significant thermodynamic variable, was only mentioned in the methane adsorption simulations to validate the reliability of the adsorption isotherm lines derived from simulation results, but its effect on the thermal conductivity was not discussed. Because the thermal conductivity calculations in this study were intended to replicate real saturated adsorption conditions, but adsorption capacities differ at various temperatures. Thus, it is not straightforward to conclude the single-variable impact on thermal conductivity with both temperature and adsorption quantity as variables. However, for pristine Cu-BTC, the thermal conductivity changes only due to a single variable, temperature, so that is convenient and logical to discuss. The PDOS of pristine Cu-BTC at 298K and 323K were examined. In the low-frequency range (0-5THz), phonon density at 298K was slightly higher than at 323K. In contrast, at other frequency ranges (5-120THz), the peaks at 298K appeared slightly compressed horizontally, making them narrower and taller compared to 323K. This indicates more regular atomic vibrations at 298K, hence a higher thermal conductivity. Besides, simulation results showed that at saturation adsorption, the thermal conductivity at 323K was lower than at 298K for all cases. Whether this can be wholly attributed to differences in MOF atoms’ vibrational regularity requires further research.

Phonon scatter, a hypothesis that adsorption reduces porous material’s thermal conductivity, was suggested by many researchers. Babaei et al. made an adsorption simulation with the pair of an idealized porous crystal (kind of simplified MOF) and methane, indicating that its thermal conductivity reduces with the methane loading rise.^40^ To a pure idealized structure, the introduction of impurity affects its original fragile thermal path, causing phonon scattering, which is reasonable intuitively. In our case, Cu-BTC is a quite complex structure, and what if the introduction of impurity just right enhances the phonon vibration, as we observed that the atomic vibration of Cu-BTC was stronger with adsorbed methane? Therefore, adsorption whether causes a kind of phonon scattering or phonon “resonance” would be an interesting topic, where mechanics can be considered as a vital role in further study. For the work of water adsorption,^26^ water, ethanol, and methanol are polar, liquid adsorbates. However, ours are non-polar, gaseous adsorbate. Besides, we discovered that the adsorbed phase of methane within Cu-BTC under the set conditions was biased toward a gaseous supercritical state. Whether the polarity or the phase of adsorbate would act as a catalyst enhancing MOF framework atomic vibrations, or as an inhibitor, is another intriguing topic. In addition, based on these hypotheses, the overall thermal conductivity for the composite adsorption system is not merely a result akin to effective medium theory,^41^ such as series or parallel outcomes, but also requires considering the properties of the adsorption pair to apply an amplifier-like term to each item.

### Conclusion

Adsorption phenomena essentially perform atomic rearrangements of both adsorbate and adsorbent. Thus, changes in their thermal conductivity are inevitable. The adsorbent, filled with adsorbate, may also conduct heat better. In this study, detailed MD simulations were employed to replicate the methane/Cu-BTC adsorption phenomenon for the evaluation of their thermal conductivities across different pressures and temperatures. The results demonstrated that due to adsorption, the free movement of methane molecules was restricted by the MOF framework, leading to reduced thermal conductivity. In contrast, the atomic vibrations of Cu-BTC were enhanced by the presence of the adsorbed methane molecules, resulting in its increased thermal conductivity. These findings are expected to be easier applied than composites, lithium doping, or nanofluids in thermal management. Future work will look at the effects of phase changes on thermal properties, potentially offering new insights into heat transfer mechanisms in nanoscale materials.

### Limitations of the study

(1) In this study, the coarse-grained methane model imposes limitations on the analysis of some results. The coarse-grained model treats the entire methane molecule as a single entity, significantly shortening simulation times and is widely applied in GCMC. However, in the analysis of thermal conductivity, this model can only calculate the overall thermal conductivity of the methane molecule and does not take into account the contribution of intra-molecular bond energy or the effect of changes in molecular alignment angles on thermal conductivity.
(2) Experimental validation of this simulation is very challenging. Separate measurement of the thermal conductivity of adsorbates within MOFs has not yet been achieved. It is hoped that changes in the vibration frequencies of atoms can be indirectly confirmed using an electron microscope.

## STAR★Methods

### Key resources table

**Table:** 

REAGENT or RESOURCE	SOURCE	IDENTIFIER
**Software and algorithms**		

LAMMPS	Open source	www.lammps.org
MATLAB	Kyushu University	https://jp.mathworks.com/academia/tah-portal/kyushu-university-31651502.html

### Resource availability

#### Lead contact

Further information and requests for resources and reagents should be directed to and will be fulfilled by the lead contact, Kyaw Thu. (kyaw.thu.813@m.kyushu-u.ac.jp)

#### Materials availability

This study did not generate new unique reagents.

#### Data and code availability

• Data reported in this paper will be shared by the lead contact upon request.
• This study does not report original code.
• Any additional information required to reanalyze the data reported in this paper is available from the lead contact upon request.

### Method details

The MD simulations were conducted using the Large-scale Atomic/Molecular Massively Parallel Simulator (LAMMPS),^42^ implementing periodic boundary conditions. The simulation encompassed four parts: adsorption of methane on Cu-BTC frameworks (Figures S1A and S1B, visualized using iRASPA^43^), and the calculation of thermal conductivity for methane-adsorbed Cu-BTC (Figure S1C), pristine Cu-BTC (Figure 1D), and the pristine methane (Figure S1E). For modeling gaseous methane, a coarse-grained TraPPE model was utilized.^44^ The Lennard-Jones (LJ) potential specific to methane, as well as the intermolecular forces between methane and Cu-BTC, were derived from the DREIDING^45^ and UFF^46^ force fields (Table S1). All the cutoff radii were set as 12.0 Å. The Lorentz-Berthelot mixing rules^32^ were applied throughout.

The force field for Cu-BTC was depicted utilizing MOF-FF,^47^ an ab initio parameterized force field tailored for MOFs. Unlike other force fields,^48^ MOF-FF incorporates the Fennell model^49^ to approximate the Ewald summation of Coulombic interactions, eliminating the need for an additional damping factor, thus streamlining the computations.

For thermal conductivity calculations, Equilibrium Molecular Dynamics (EMD) and Non-Equilibrium Molecular Dynamics (NEMD) represent the two principal methodologies.^50^ The EMD approach, employed in this study, does not necessitate a heat source or sink, offering greater flexibility in terms of box configuration. The thermal conductivity calculations were performed based on the Green-Kubo (GK) method.^51^ Furthermore, to validate the GK method, the thermal conductivity calculated by Einstein relation for the pristine methane was utilized as a reference (Figure 1D). The solver for Einstein relation was facilitated by the OCTP plugin, as developed by Jamali S.H. et al..^33^

The reliability of the results was ascertained by averaging over four independent simulation runs for each scenario.

#### Methane adsorption simulations

The simulation study delineated the methane adsorption dynamics on Cu-BTC under isothermal and isobaric conditions. This process comprised two steps: the ambient methane creation (Figure 1A) and its subsequent adsorption (Figure 1B). Initially, a simulation box with a cubic length of 400 Å was populated with methane molecules to mimic a gaseous ambient phase. The molecular count corresponded to the gas’s density at five distinct pressures, 5, 10, 15, 20, and 25 bar, and at two temperatures, 298K and 323K. A time step of 1 fs was maintained. Equilibration proceeded for 1 ns within the NVT ensemble, utilizing a Nosé-Hoover thermostat for temperature regulation,^52^ followed by a 2 ns production run in the NVE ensemble. The thermal parameters, namely temperature and pressure, were correlated with the methane density using the reference data from REFPROP/NIST ver. 10.^53^ This high-density methane environment was established to ensure the pressure remained essentially constant during the adsorbed phase.

Subsequently, a Cu-BTC framework, with a density of 869.38 kg/m^3^, assembled from 2 × 2 × 2 unit cells and measuring 52.8816 Å per side, was centrally embedded within the methane box and froze. To avoid the effect of the periodic boundary of Cu-BTC due to its configuration file, all bonds and angles information was deleted temporarily (Added again in subsequence simulations). Methane molecules within the immediate vicinity of the Cu-BTC were removed to set the initial adsorption at zero. The adsorption simulation spanned 5 ns in the NVT ensemble, except for the 5 and 10 bar conditions, which were extended to 8 ns to accommodate equilibration. The methane uptake by the Cu-BTC was monitored by counting the number of methane molecules within the Cu-BTC region. Results were derived by averaging the uptake across four independent simulation trials to ensure the robustness of the findings.

#### Methane/Cu-BTC thermal conductivity simulation

The simulation of the Cu-BTC with adsorbed methane aimed to ascertain the thermal conductivity of the composite system as well as its components. This simulation commenced from the final state of the preceding methane adsorption simulation. The procedure involved several key steps. Initially, ambient methane molecules were removed, and the simulation box dimensions were modified to correspond to the size of the cubic Cu-BTC (from Figures 1B and 1C). Subsequently, the frozen Cu-BTC framework was ‘unlocked’ and distributed with MOF-FF. Then the velocities of all molecules were reinitialized, a critical step to ensure the correct dynamic correlation.

The simulation proceeded with a time step of 1 fs and included a relaxation period of 1 ns within the NVT ensemble, under the temperature control of a Nosé-Hoover thermostat and followed by a 10 ns run in the NVE ensemble, with the latter half designated for production.

During the simulation, three Green-Kubo calculators operated in tandem to evaluate the thermal conductivities of the entire system, the Cu-BTC component, and the methane component within the system, respectively. The Green-Kubo calculations utilized a correlation time of 500 ps to determine the thermal conductivity.

The robustness and accuracy of the simulation results were enhanced by averaging outcomes from four independent simulation runs for each condition. Additionally, for each simulation, 10 data profiles were generated to facilitate a comprehensive analysis.

#### Cu-BTC thermal conductivity simulation

In the simulation dedicated to evaluating the thermal conductivity of pristine Cu-BTC, the same Cu-BTC framework configuration was employed, consisting of 2 × 2 × 2 unit cells within a cubic space in length of 52.8816 Å (Figure 1D). The simulation parameters, including the time step, relaxation process, and production run, were kept consistent with the methodology applied in the methane/Cu-BTC thermal conductivity simulation. The only distinction was the utilization of a single Green-Kubo calculator, which was tasked with calculating the thermal conductivity of the pristine Cu-BTC framework exclusively.

#### Methane thermal conductivity simulation

The objective of this simulation was to determine the thermal conductivity of pristine methane in the adsorbed phase. The phase state of methane during adsorption, either gas or liquid, dependeds on its interaction with a particular adsorbent. In this study, the number of methane molecules was determined from the methane/Cu-BTC thermal conductivity simulation (Figure 1E).

Two approaches were adopted to evaluate the thermal conductivity: the Green-Kubo method and the Einstein relation method. The Green-Kubo simulations were performed with the same box size, and simulation parameters as those used for the Cu-BTC thermal conductivity simulations.

In contrast, the Einstein method simulations followed a similar protocol for the box size and the time step, which was set to 1 fs. The system was relaxed for 1 ns within an NVT ensemble, with temperature regulation achieved through a Nosé-Hoover thermostat, followed by a 2 ns production phase within the NVE ensemble. The Einstein method simulations benefited from the order-N algorithm,^54^ which allowed for a more expeditious completion time.

#### Auxiliary simulations

To elucidate the mechanisms governing thermal conductivity at the microscale during the adsorption phenomena, three auxiliary simulations were performed: the mean free path (MFP) calculation for methane in both the methane/Cu-BTC composite system and pristine methane, the lattice mean displacement (LMD) calculation for Cu-BTC in the methane/Cu-BTC combined system as well as pristine Cu-BTC, and the phonon density of states (PDOS) calculation for both the methane/Cu-BTC composite system and pristine Cu-BTC.

The MFP and LMD calculations served to qualitatively assess the variations in thermal conductivity for methane and Cu-BTC, respectively. These calculations were performed on the molecular coordinates, which were extracted from the NVE ensemble over a 1 ns duration, sampling every 10 timesteps. Before the NVE, 1 ns of NVT relaxation with a timestep of 1 fs was proceeded. MFP is the average distance a molecule particle travels between successive collisions with other particles. As shown in Figure S2A, the collision was judged if the distance of two molecules was close to the LJ distance parameter, σ. The red trajectory represents the free path between successive collisions, and the MFP was calculated by Equation 1:

(Equation 1) $$\text{MFP}=\frac{\sum_{i}l}{\sum_{i}C}$$

Here, *l* is the total traveled distance of one molecule during simulation time, and C is the number of collisions. LMD refers to the mean displacement of each atom in Cu-BTC from its initial position during the simulation, depicted in Figure S2B, providing insight into the intensity of atomic vibrations, according to Equation 2:

(Equation 2) $$\text{LMD}=\frac{\sum_{i}\left|r_{t}-r_{0}\right|}{N}$$

Here *r* is the vector of atom coordinate, *N* is the number of atoms. MFP and LMD were carried out on the platform of MATLAB.

The Phonon Density of States (PDOS) calculation was designed to characterize the frequency distribution of phonons within the system, which is crucial for understanding the thermal behavior at the atomic level.^55^ The calculation utilized the molecular velocities that were recorded every timestep during a 100 ps in NVE ensemble, after 1 ns of NVT and 1 ns of NVE relaxation periods, with the timestep of 1 fs. A correlation time of 5 ps was selected to ensure the convergence of the velocity autocorrelation function (VACF). The PDOS is expressed in Equation 3:

(Equation 3) $$PDOS=\int \frac{<\sum_{i}\;v_{i}\left(t_{0}\right)\cdot v_{i}\left(t_{0}+t\right)>}{<\sum_{i}\;v_{i}\left(t_{0}\right)\cdot v_{i}\left(t_{0}\right)>}e^{-2\pi i\omega t}dt$$

where *v* is the velocity for the molecule, and ω is the frequency of the targeted phonon, which ranges from 0 to 120 THz. The PDOS were implemented via scripting in Python.

### Quantification and statistical analysis

Apart from the mean free path and the lattice mean displacement, which are calculated using MATLAB, all other post-processing calculations are performed using Python.

## References

[1] Wang X., Song C. (2020). Carbon Capture From Flue Gas and the Atmosphere: A Perspective. Front. Energy Res..

[2] Abouelella D.M., Fateen S.E.K., Fouad M.M. (2018).

[3] Pinheiro J.M., Salústio S., Rocha J., Valente A.A., Silva C.M. (2020). Adsorption heat pumps for heating applications. Renew. Sustain. Energy Rev..

[4] Rouf R.A., Khan M.A., Kabir K.M., Saha B.B. (2016).

[5] Vivekh P., Kumja M., Bui D.T., Chua K.J. (2018). Recent developments in solid desiccant coated heat exchangers – A review. Appl. Energy.

[6] Karmakar A., Prabakaran V., Zhao D., Chua K.J. (2020). A review of metal-organic frameworks (MOFs) as energy-efficient desiccants for adsorption driven heat-transformation applications. Appl. Energy.

[7] Elsayed E., AL-Dadah R., Mahmoud S., Anderson P., Elsayed A. (2020). Experimental testing of aluminium fumarate MOF for adsorption desalination. Desalination.

[8] Moayed Mohseni M., Jouyandeh M., Mohammad Sajadi S., Hejna A., Habibzadeh S., Mohaddespour A., Rabiee N., Daneshgar H., Akhavan O., Asadnia M. (2022). Metal-organic frameworks (MOF) based heat transfer: A comprehensive review. Chem. Eng. J..

[9] Islamov M., Babaei H., Anderson R., Sezginel K.B., Long J.R., McGaughey A.J.H., Gomez-Gualdron D.A., Wilmer C.E. (2023). High-throughput screening of hypothetical metal-organic frameworks for thermal conductivity. npj Comput. Mater..

[10] Gunatilleke, W.D.C. B., Wei K., Niu Z., Wojtas L., Nolas G., Ma S. (2017). Thermal conductivity of a perovskite-type metal–organic framework crystal. Dalton Trans..

[11] Huang B.L., Ni Z., Millward A., McGaughey A.J.H., Uher C., Kaviany M., Yaghi O. (2007). Thermal conductivity of a metal-organic framework (MOF-5): Part II. Measurement. Int. J. Heat Mass Transf..

[12] Cui B., Audu C.O., Liao Y., Nguyen S.T., Farha O.K., Hupp J.T., Grayson M. (2017). Thermal Conductivity of ZIF-8 Thin-Film under Ambient Gas Pressure. ACS Appl. Mater. Interfaces.

[13] Huang B.L., McGaughey A.J.H., Kaviany M. (2007). Thermal conductivity of metal-organic framework 5 (MOF-5): Part I. Molecular dynamics simulations. Int. J. Heat Mass Transf..

[14] Zhang X., Jiang J. (2013). Thermal Conductivity of Zeolitic Imidazolate Framework-8: A Molecular Simulation Study. J. Phys. Chem. C.

[15] Wieme J., Vandenbrande S., Lamaire A., Kapil V., Vanduyfhuys L., Van Speybroeck V. (2019). Thermal Engineering of Metal–Organic Frameworks for Adsorption Applications: A Molecular Simulation Perspective. ACS Appl. Mater. Interfaces.

[16] Islamov M., Babaei H., Wilmer C.E. (2020). Influence of Missing Linker Defects on the Thermal Conductivity of Metal–Organic Framework HKUST-1. ACS Appl. Mater. Interfaces.

[17] Babaei H., McGaughey A.J.H., Wilmer C.E. (2017). Effect of pore size and shape on the thermal conductivity of metal-organic frameworks. Chem. Sci..

[18] Huang J., Xia X., Hu X., Li S., Liu K. (2019). A general method for measuring the thermal conductivity of MOF crystals. Int. J. Heat Mass Transf..

[19] Cui S., Marandi A., Lebourleux G., Thimon M., Bourdon M., Chen C., Severino M.I., Steggles V., Nouar F., Serre C. (2019). Heat properties of a hydrophilic carboxylate-based MOF for water adsorption applications. Appl. Therm. Eng..

[20] Han L., Budge M., Alex Greaney P. (2014). Relationship between thermal conductivity and framework architecture in MOF-5. Comput. Mater. Sci..

[21] Wei W., Huang J., Li W., Peng H., Li S. (2019). Impacts of Ethanol and Water Adsorptions on Thermal Conductivity of ZIF-8. J. Phys. Chem. C.

[22] Babaei H., Lee J.-H., Dods M.N., Wilmer C.E., Long J.R. (2020). Enhanced Thermal Conductivity in a Diamine-Appended Metal–Organic Framework as a Result of Cooperative CO_2_ Adsorption. ACS Appl. Mater. Interfaces.

[23] Giri A., Hopkins P.E. (2021). Heat Transfer Mechanisms and Tunable Thermal Conductivity Anisotropy in Two-Dimensional Covalent Organic Frameworks with Adsorbed Gases. Nano Lett..

[24] Wang H., Qu Z.G., Yin Y., Bai J.Q., He C. (2021). Prediction of the effective thermal conductivity of an adsorption bed packed with 5A zeolite particles under working conditions. Int. J. Therm. Sci..

[25] Jiang L., Roskilly A.P. (2019). Thermal conductivity, permeability and reaction characteristic enhancement of ammonia solid sorbents: A review. Int. J. Heat Mass Transf..

[26] Babaei H., DeCoster M.E., Jeong M., Hassan Z.M., Islamoglu T., Baumgart H., McGaughey A.J.H., Redel E., Farha O.K., Hopkins P.E. (2020). Observation of reduced thermal conductivity in a metal-organic framework due to the presence of adsorbates. Nat. Commun..

[27] Fan H., Yang C., Zhou Y. (2022). Ultralong mean free path phonons in HKUST-1 and their scattering by water adsorbates. Phys. Rev. B.

[28] Purewal J.J., Liu D., Yang J., Sudik A., Siegel D.J., Maurer S., Müller U. (2012). Increased volumetric hydrogen uptake of MOF-5 by powder densification. Int. J. Hydrogen Energy.

[29] Schlemminger C., Næss E., Bünger U. (2015). Adsorption hydrogen storage at cryogenic temperature – Material properties and hydrogen ortho-para conversion matters. Int. J. Hydrogen Energy.

[30] Liang Z., Marshall M., Chaffee A.L. (2009). CO_2_ Adsorption-Based Separation by Metal Organic Framework (Cu-BTC) versus Zeolite (13X). Energy Fuels.

[31] Aimoli C.G., Maginn E.J., Abreu C.R.A. (2014). Transport properties of carbon dioxide and methane from molecular dynamics simulations. J. Chem. Phys..

[32] Brooks C.L. (1989). Computer simulation of liquids. J. Solution Chem..

[33] Jamali S.H., Wolff L., Becker T.M., de Groen M., Ramdin M., Hartkamp R., Bardow A., Vlugt T.J.H., Moultos O.A. (2019). OCTP: A Tool for On-the-Fly Calculation of Transport Properties of Fluids with the Order-n Algorithm in LAMMPS. J. Chem. Inf. Model..

[34] Surblys D., Matsubara H., Kikugawa G., Ohara T. (2021). Methodology and meaning of computing heat flux via atomic stress in systems with constraint dynamics. J. Appl. Phys..

[35] Dubbeldam D., Calero S., Ellis D.E., Snurr R.Q. (2016). RASPA: molecular simulation software for adsorption and diffusion in flexible nanoporous materials. Mol. Simul..

[36] Skoulidas A.I. (2004). Molecular Dynamics Simulations of Gas Diffusion in Metal−Organic Frameworks: Argon in CuBTC. J. Am. Chem. Soc..

[37] Cheng R., Wei W., Zhang J., Li S. (2023). Understanding the Heat Transfer Performance of Zeolitic Imidazolate Frameworks upon Gas Adsorption by Molecular Dynamics Simulations. J. Phys. Chem. B.

[38] Fan H., Ying P., Fan Z., Chen Y., Li Z., Zhou Y. (2024). Anomalous strain-dependent thermal conductivity in the metal-organic framework HKUST-1. Phys. Rev. B.

[39] Qian X., Zhou J., Chen G. (2021). Phonon-engineered extreme thermal conductivity materials. Nat. Mater..

[40] Babaei H., Wilmer C.E. (2016). Mechanisms of Heat Transfer in Porous Crystals Containing Adsorbed Gases: Applications to Metal-Organic Frameworks. Phys. Rev. Lett..

[41] Choy T.C. (2015).

[42] Thompson A.P., Aktulga H.M., Berger R., Bolintineanu D.S., Brown W.M., Crozier P.S., in ’t Veld P.J., Kohlmeyer A., Moore S.G., Nguyen T.D. (2022). LAMMPS - a flexible simulation tool for particle-based materials modeling at the atomic, meso, and continuum scales. Comput. Phys. Commun..

[43] Dubbeldam D., Calero S., Vlugt T.J.H. (2018). iRASPA: GPU-accelerated visualization software for materials scientists. Mol. Simul..

[44] Martin M.G., Thompson A.P., Nenoff T.M. (2001). Effect of pressure, membrane thickness, and placement of control volumes on the flux of methane through thin silicalite membranes: A dual control volume grand canonical molecular dynamics study. J. Chem. Phys..

[45] Mayo S.L., Olafson B.D., Goddard W.A. (1990). DREIDING: a generic force field for molecular simulations. J. Phys. Chem..

[46] Rappe A.K., Casewit C.J., Colwell K.S., Goddard W.A., Skiff W.M. (1992). UFF, a full periodic table force field for molecular mechanics and molecular dynamics simulations. J. Am. Chem. Soc..

[47] Bureekaew S., Amirjalayer S., Tafipolsky M., Spickermann C., Roy T.K., Schmid R. (2013). MOF-FF – A flexible first-principles derived force field for metal-organic frameworks. Phys. Status Solidi.

[48] Raabe G., Maginn E.J. (2010). A Force Field for 3,3,3-Fluoro-1-propenes, Including HFO-1234yf. J. Phys. Chem. B.

[49] Fennell C.J., Gezelter J.D. (2006). Is the Ewald summation still necessary? Pairwise alternatives to the accepted standard for long-range electrostatics. J. Chem. Phys..

[50] Matsubara H., Kikugawa G., Ishikiriyama M., Yamashita S., Ohara T. (2017). Equivalence of the EMD- and NEMD-based decomposition of thermal conductivity into microscopic building blocks. J. Chem. Phys..

[51] Searles D.J., Evans D.J. (2000). The fluctuation theorem and Green–Kubo relations. J. Chem. Phys..

[52] Evans D.J., Holian B.L. (1985). The Nose–Hoover thermostat. J. Chem. Phys..

[53] Huber M.L., Lemmon E.W., Bell I.H., McLinden M.O. (2022). The NIST REFPROP Database for Highly Accurate Properties of Industrially Important Fluids. Ind. Eng. Chem. Res..

[54] Dubbeldam D., Ford D.C., Ellis D.E., Snurr R.Q. (2009). A new perspective on the order-n algorithm for computing correlation functions. Mol. Simul..

[55] DICKEY J.M., PASKIN A. (1969). Computer Simulation of the Lattice Dynamics of Solids. Phys. Rev..

