# Supplementary material for: Impact of adsorption on thermal conductivity dynamics of adsorbate and adsorbent: Molecular dynamics study of methane and Cu-BTC
Source: iScience. 2024 Jul 4;27(8):110449. doi: 10.1016/j.isci.2024.110449 (PMC11298659; doi:10.1016/j.isci.2024.110449)
Supplement: Document S1. Figures S1 and S2 and Table S1 [file mmc1.pdf]

iScience, Volume *27*

Supplemental information

Impact of adsorption on thermal conductivity dynamics of adsorbate and adsorbent: Molecular dynamics study of methane and Cu-BTC

Haonan Chen, Sagar Saren, Xuetao Liu, Ji Hwan Jeong, Takahiko Miyazaki, Young-Deuk Kim, and Kyaw Thu

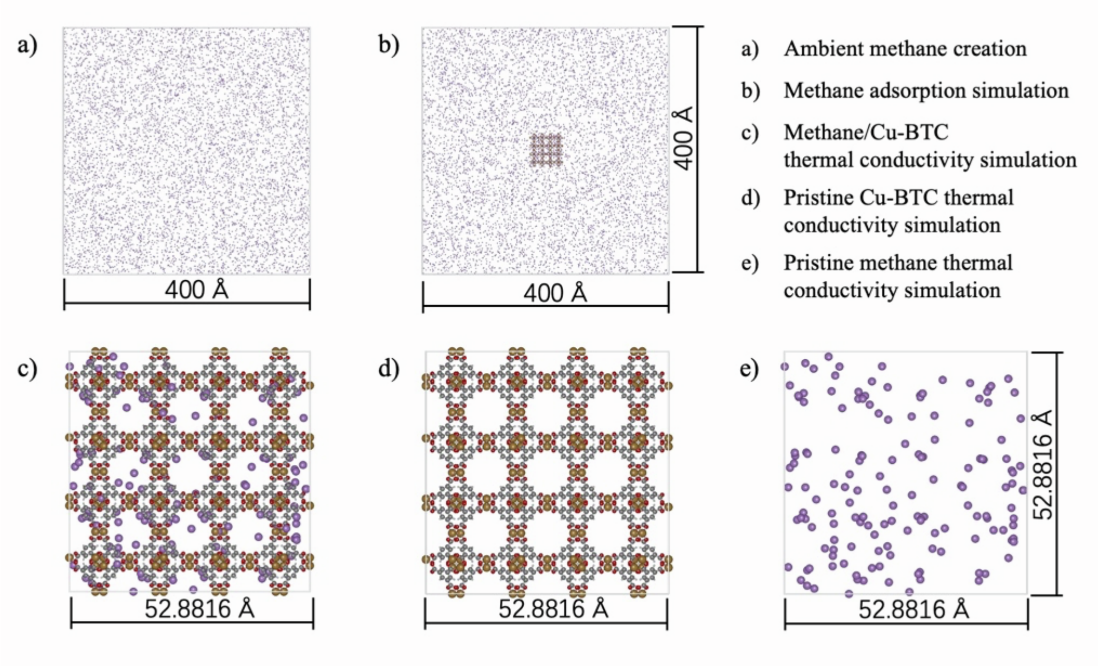

Figure S1: Simulation configurations, related to STAR Methods.
(a) Ambient methane creation. (b) Methane adsorption simulation. (c) Methane/Cu-BTC thermal conductivity simulation. (d) Pristine Cu-BTC thermal conductivity simulation. (e) Pristine methane thermal conductivity simulation.

Table S1: LJ parameter for interaction force of methane and Cu-BTC, related to STAR Methods.

**Atom**	**ε (kcal/mol)**	**σ (Å)**
CH_4-C1	0.1672	3.60
CH_4-C2	0.1672	3.60
CH_4-C3	0.1672	3.60
CH_4-Cu	0.0383	3.42
CH_4-O	0.1678	3.38
CH_4-H	0.0669	3.28
CH_4-CH_4	**0.2941**	**3.72**

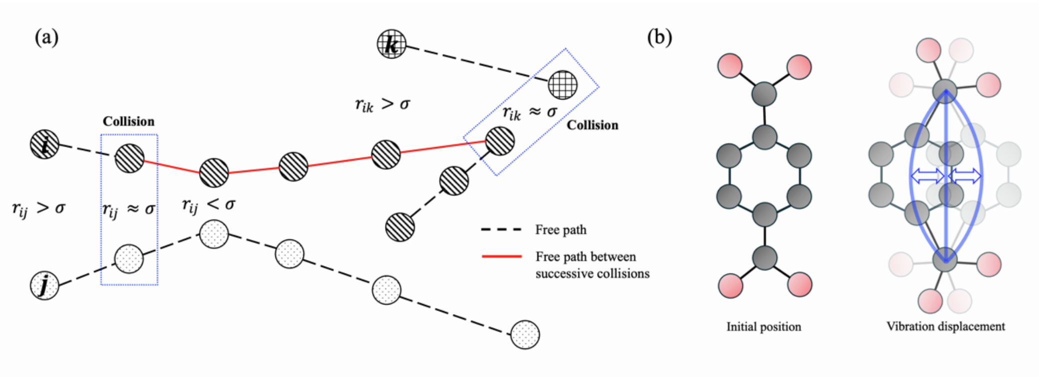

Figure S2: The schematic of auxiliary simulation, related to STAR Methods.
(a) mean free path calculation. (b) lattice mean displacement.
